# Pulmonary tuberculosis and rhinosinus mucormycosis co-infection in a diabetic patient

**DOI:** 10.18502/cmm.8.2.10332

**Published:** 2022-06

**Authors:** Shiva Shabani, Payam Tabarsi, Golnaz Afzal

**Affiliations:** 1 Department of Infectious Diseases, School of Medicine, Arak University of Medical Sciences, Arak, Iran; 2 Department of Infectious Diseases, School of Medicine, Ayatollah Khansari Hospital, Arak University of Medical Sciences, Arak, Iran; 3 Clinical Tuberculosis and Epidemiology Research Center, National Research Institute of Tuberculosis and Lung Diseases (NRITLD), Shahid Beheshti University of Medical Sciences, Tehran, Iran; 4 Department of Clinical Pharmacy, School of Pharmacy, Shahid Beheshti University of Medical Sciences, Tehran, Iran

**Keywords:** Diabetes, Mucormycosis, Pulmonary tuberculosis

## Abstract

**Background and Purpose:**

Diabetes and immunosuppressive diseases have been reported as increased risk factors for developing invasive pulmonary tuberculosis and mucormycosis.

**Case report:**

We presented here a case of a 55-year-old uncontrolled diabetic male with rhinosinus mucormycosis and pulmonary TB coinfection. Maxillary and ethmoid sinus involvement was observed in paranasal computed tomography. His chest computed tomography showed tree in the bud sign and cavitary lesions in the lungs. *Mycobacterium tuberculosis* was confirmed through molecular diagnosis using a real-time polymerase chain reaction assay. The nasal cavity biopsy revealed the fungal elements (aseptate hyphae) and confirmed mucormycosis infection. Amphotericin B liposomal, teicoplanin, and tazobactam were administered to treat the mucormycosis. The patient was successfully treated with a recommended four-drug regimen for TB without any adverse reaction.

**Conclusion:**

The clinicians must consider tuberculosis and mucormycosis tests when confronted with an uncontrolled diabetic patient with clinical symptoms of hemoptysis, fever, and cavitary lesions

## Introduction

Mucormycosis is an emerging opportunistic fungus causing the development of invasive infection, especially in immunocom-promised individuals and those with uncontrolled diabetes [ 1 , 2 ]. Mucormycosis has a high mortality rate (50- 80%) in high-risk patient groups including patients suffering from uncontrolled diabetes, metabolic acidosis, malignancies, renal failure, long-term immunosuppressive treatment, and transplant recipients [ 3 - 5 ].

About half of mucormycosis infections occur in patients with uncontrolled diabetes due to tooth removal, or ophthalmic surgeries [ 6 ]. Inhalation of mucor spores and its spread to the sino-orbital or rhinocerebral region leads to infection of the paranasal sinus [ 6 , 7 ]. Rhinocerebral mucormycosis is a commonly reported (30 - 50%) infection and usually progresses to involve the orbit and brain [ 7 ]. Diabetes and immunosuppressive diseases have been reported as increased risk factors for developing invasive pulmonary tuberculosis (TB) globally [ 1 , 2 , 8 , 9 ]. The mortality rate of TB in developing countries is more than 90% and 23% of whom have latent TB infection [ 3 ]. An estimated one million patients around the world are diagnosed with active TB- Diabetes coinfection [ 10 ]. Mucormycosis infections are detected with histopathological assessment and TB is diagnosed based on a combination of clinical features, imaging findings, and molecular detection [ 3 ]. This study aimed to report a case of a diabetic patient with rhinosinus mucormycosis and pulmonary TB, emphasizing the need for more attention. Successful treatment of such a co-existing disease has not been reported in our country (Iran) to date.

## Case report

In April 2019, a 55- year-old man with uncontrolled diabetes was referred to Masih Daneshvari Hospital, Tehran, Iran, with complaints of fever, weakness, productive cough, and occasional hemoptysis for three months. He was addicted to cannabis for 20 years and had experienced localized one-side facial pain and swelling seven days after dental extraction. 

**Figure 1:**
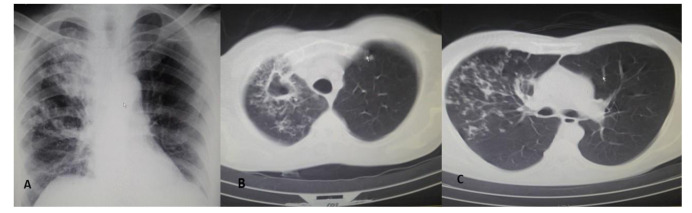
Chest radiography of posterior-anterior projection (view) and thorax computed tomography A. Chest X-ray, posterior-anterior projection, right upper lung infiltration upon admission B. Computed tomography of the right lung cavitary lesions in the right lung C. Computed tomography of the right lower lung showing tree-in-bud infiltration infiltrations

 In this recumbent patient, the Glasgow coma scale was determined as 15 without focal neurologic shortage or meningeal signs, dehydrated skin and mucosal membrane, and awareness of his environment. His medical history showed that he had uncontrolled diabetes for many years with fasting blood sugar level, weight, and height of 273 mg/dL, 70 kg, and 170 cm, respectively. His chest examination and chest x-ray were normal (intensity, pitch, duration, and chest percussion quality). Precordial auscultation showed normal heart sounds, and abdominal exploration was normal as well. The chest CT imaging showed ground‑glass opacities in the right lower lung in both lungs Vital signs and the laboratory results at the time of admission are presented in Table 1. Upon hospital admission, the chest radiography of posterior-anterior projection view revealed right upper lobe alveolar infiltration (Figure 1a). A thorax computed tomography (CT) showed tree-in-bud infiltration infiltrations in the right lower lung (Figure 1b); (Figure 1c). 

**Table 1:** Abnormal clinical laboratory results and vital signs of the presented case at first admission

Test	Reference range	Value
Hemoglobin A1c (%)	4-5.6%	11.7%
Blood urea nitrogen (mg/dL)	8.4-25.7	28
Creatinine (mg/dL)	0.9-1.4	1.0
Alanine aminotransferase (U/liter)	10-33	51
Erythrocyte sedimentation rate (mm)	0-20	117
Fasting blood sugar (FBS) (mg/dL)	60-99	273
**Vital sings**	**Normal range**	**Value**
Temperature	36.1°C to 37.9 °C	38.1 °C
PO_2_	35 to 45 mmHg	28 mmHg

* White blood cells, hemoglobin, platelets, creatinine, aspartate aminotransferase, lactic acid dehydrogenase, and uric acid levels and vital signs including blood pressure, heart rate, respiratory rate, venous blood test gas test, carbon dioxide gas pressure in the blood, or bicarbonate were in normal ranges.

The early antibiotic regimen included liposomal amphotericin B, piperacillin, tazobactam, and teicoplanin. Due to the high likelihood of mucormycosis rhinosinusitis, nasal sinus endoscopy was performed with a positive result. Considering the high probability of pulmonary tuberculosis, a direct smear was prepared from sputum and induced sputum samples. 

**Figure 2:**
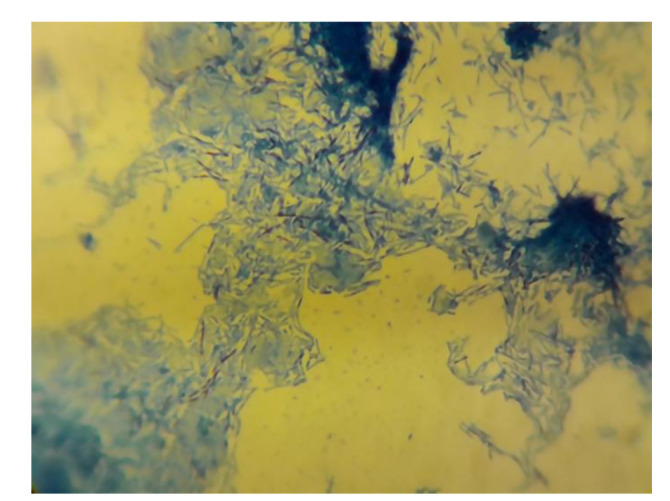
Acid-resistant bacilli stained bright red on a blue background compatible with M. tuberculosis from sputum specimens

Acid-fast *bacillus* was detected from three frequency sputum specimens (Figure 2) and the presence of *M. tuberculosis* mycobacterial DNA was confirmed in molecular diagnosis using real-time polymerase chain reaction (RT-PCR). In brief, the specimen was cultured in a Lowenstein-Jensen medium, and DNA was extracted using the QIAamp DNA Mini Kit (Qiagen, Germany). A quantitative RT-PCR assay was conducted using a Rotor-Gene 6000 RT-PCR cycler (Qiagen Corbett, Hilden, Germany) and PCR kit qPCR master mix (QuantiTect SYBR® Green; Yekta Tajhiz Azuma, Tehran, Iran). 

**Figure 3:**
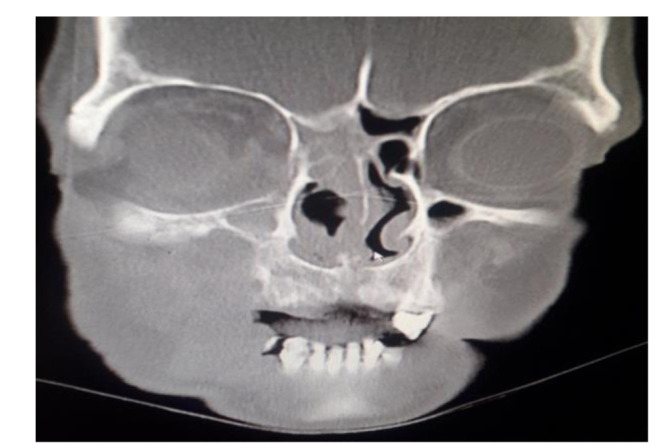
Paranasal sinus radiograph showing involvement of the right nasal cavity with the destruction of sinus walls

In the second week of hospitalization, angioembolisation was performed and the histopathological report of functional endoscopic sinus surgery from the nasal cavity revealed respiratory-type mucosa with necroinflammation and the presence of fragmented and thick hyaline fungal hyphae, compatible with Mucorales Mucoral species (Figure 3).

**Figure 4:**
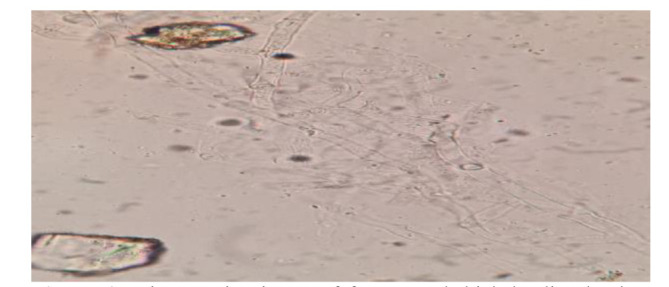
Microscopic picture of fragmented thick hyaline hyphae (mucormycosis) treated with KOH 10%(×400)

In addition, a biopsy of the nasal cavity ( prepared in KOH wet mount) confirmed mucormycosis by the presence of fungal elements (aseptate hyphae) (Figure 4). 

A final diagnosis of mucormycosis and tuberculosis coinfection was made. The treatment was initiated with the administration of rifampicin 600 mg PO q24 h, isoniazid 300 mg PO q24 h, pyrazinamide 1500 mg PO q24 h, and ethambutol 1200 mg PO q24 h, followed by liposomal administration of Amphotericin B (5 mg/kg/d for 3 weeks). His rhinosinus mucormycosis was treated with a total dose of 6.5 g tazobactam 4.5g q 8 h teicoplanin 400mg q1 2 h for three doses and then 400 mg daily. Paranasal sinus endoscopy was performed. Ophthalmology consultation was also performed several times. 

The patient was discharged 30 days after admission due to clinical improvement. Directly observed therapy was continued and oral posaconazole (400 mg bid) was administered as an antifungal. No histopathological findings of sinus mucormycosis were reported in the last surgical endoscopic piece biopsy report.

## Discussion

Opportunistic infections are common forms of infections in patients with uncontrolled diabetes [ 4 ]. Concomitant rhinosinus mucormycosis and pulmonary TB is a rare and potentially fatal disease with a poor prognosis [ 6 , 7 ]. 

Coinfection of mucormycosis and TB was successfully treated in our non- ketoacidosis diabetic case. The patient responded well to the treatments for TB and rhinosinus mucormycosis. Other studies showed that the poor outcome of this coinfection may be the consequence of a late diagnosis. Diabetes leads to immune dysfunction and flare-up of mucormycosis coinfection with latent TB which may progress to active TB and fulminant outcome. Clinical manifestations include fever, consolidation, or nodules [ 3 , 11 ]. 

Previous studies reported only nine concomitant mucormycosis and TB in England, Japan, and India [ 1 , 3 , 12 , 13 ]. This has been the first report of an uncontrolled diabetic case with rhinosinus mucormycosis and pulmonary TB coinfection in our country. Uncontrolled diabetic patients are at higher risk of developing rhinocerebral mucormycosis related to abnormal phagocytic activity and immunity [ 6 ]. In this patient with focal neurologic shortage, the necrotic area was observed in the right sinuses, indicating the severity and extent of the disease. The tooth extraction and the dental instrument could be the sources of infection. The presence of extensive infection was observed and confirmed in the facial CT scans. Other studies have reported that tooth extraction is an important risk factor for the development of severe fungal infections in patients with poorly controlled diabetes [ 1 , 6 ]. Other risk factors include immunosuppression due to organ transplant, long-term corticosteroid or immunosuppressive treatment, and hemochromatosis [ 7 ]. At the initial stage of the disease, imaging methods are not useful due to the thickness of the sinus mucosa [ 7 ], and CT or MRI scans should be performed based on the progression of the disease and appropriate surgical interventions [ 4 , 7 ]. In the present case, the CT scan showed the extent of nasal destruction, and histopathologically, the lesion demonstrated fragmented thick hyaline fungal hyphae of Mucorales Mucoral species. Moreover, due to the vaso-occlusive nature of the infection, the initial antibiotic management consisted of liposomal Amphotericin B (IV). Hyperglycemia leads to dysfunctional innate and adaptive immunity, which in turn increases the risk of various opportunistic infections such as tuberculosis, mucormycosis, and other fungal proliferation [ 2 , 4 , 7 , 14 ].

In Asian people, poor glycemic control (as indicated by HbA1c level) represents a potentially important risk factor for active TB [ 10 ]. Diabetes mellitus in developing countries will rise to 366 million worldwide by 2030. Therefore, individuals with important risk factors for TB in our region should be screened and diabetes mellitus control programs should be launched for different populations, particularly young people and TB patients [ 9 ]. Late diagnosis of TB in a tuberculosis endemic country may be due to the high prevalence of nonspecific symptoms of pulmonary tuberculosis [ 1 , 6 ].

The high mortality of TB coinfection and opportunistic fungal pathogens, such as mucormycosis, necessitates a high degree of clinical suspicion, definitive diagnosis, and early aggressive treatment [ 11 ].

The chest X-ray and the thorax CT of our case suggested TB, a smear of sputum that indicated the presence of acid-fast bacillus (AFB). *M. tuberculosis* mycobacterial ribosomal RNA was observed as well using RT-PCR. Moreover, fragmented thick hyaline fungal hyphae confirmed mucormycosis on culture. Therefore, mucormycosis and TB were successfully treated.

It should be noted that the poor outcome of other similar studies may be the consequence of a late diagnosis due to the focus on treating diabetic ketoacidosis [ 11 ]. The recommended antifungal therapy (amphotericin B liposomal, teicoplanin, and tazobactam) and surgical debridement were used until the normalization of X-ray findings and negative culture [ 1 ]. Our patient was successfully treated with a recommended four-drug regimen for TB treatment without any adverse reaction. 

Although identification of the fungi to the species level is essential for further proper treatment, the lack of specific- species identification of the recovered isolate from the patient posed a limitation to this study.

## Conclusion

This potentially fatal opportunistic fungal infection remains a poorly understood disease. The case presented here was associated with *Mycobacterium tuberculosis* and rhinosinus mucormycosis coinfection in a patient with uncontrolled diabetes. The clinicians must consider tuberculosis and mucormycosis tests when confronted with an uncontrolled diabetic patient with clinical symptoms of hemoptysis, fever, and cavitary lesions.

## Acknowledgments

We would like to thank the staff of Masih Daneshvari Hospital for their special co-operation in this study.

## Authors’ contribution

SH. SH. and T. P. contributed to the experimental studies, diagnosis, and treatment of the case, T. P., A.G, and SH. SH. contributed to discussion, implications, analysis of the data, and preparation of the manuscript. All authors read and approved the final manuscript. 

## Conflicts of interest

The authors declared that they have no competing interests.

## Financial disclosure

None.

## Ethical Considerations

This case report was approved by the human research ethics committee of Shahid Beheshti University of Medical Sciences, Tehran, Iran (IR.SBMU.REC 1398.809).
